# Short-term impact of COVID-19 pandemic on low back pain: data from the PAMPA Cohort, Brazil

**DOI:** 10.1186/s12889-022-14932-w

**Published:** 2023-01-06

**Authors:** Eduardo L. Caputo, Paulo H. Ferreira, Natan Feter, Igor R. Doring, Jayne S. Leite, Ricardo Alt, Júlia Cassuriaga, Felipe F. Reichert, Airton J. Rombaldi, Marcelo C. da Silva

**Affiliations:** 1 Postgraduate Program in Physical Education, Federal University of Pelotas, Pelotas, Brazil; 2 Neuroscience and Physical Activity Research Group, Federal University of Pelotas, Pelotas, Brazil; 3 Discipline of Physiotherapy, Faculty of Health Sciences, The University of Sydney, Sydney, NSW Australia; 4 Postgraduate Program in Epidemiology, Federal University of Rio Grande Do Sul, Porto Alegre, Brazil; 5 School of Human Movement and Nutrition Sciences, The University of Queensland, St Lucia, Australia; 6 Postgraduate Program in Cardiology and Cardiovascular Sciences, Federal University of Rio Grande Do Sul, Porto Alegre, Brazil; 7 Postgraduate Program in Epidemiology, Federal University of Pelotas, Pelotas, Brazil

**Keywords:** Low back pain, COVID-19, Physical activity

## Abstract

**Background:**

To evaluate the short-term impact of COVID-19 pandemic on low back pain (LBP) outcomes in southern Brazil.

**Methods:**

Data from the PAMPA Cohort were analyzed. Adults were recruited between June and July 2020 in the Rio Grande do Sul state using online-based strategies. Participants responded a self-reported, online questionnaire on LBP with two timepoints: before (retrospectively) and during COVID-19 pandemic. We assessed LBP experience, LBP-related activity limitation (no/yes), and LBP intensity (0 to 10 [strongest pain]).

**Results:**

From a total sample of 2,321 respondents (mean age: 37.6 ± 13.5; 75.4% women), the prevalence of LBP did not change significantly from before (74.7% [95%CI 72.3; 76.9]) to the first months of pandemic (74.2% [95%CI 71.9; 76.3]). However, an increased pain levels (β: 0.40; 95%CI 0.22; 0.58) and a higher likelihood for activity limitation due to LBP was observed (PR 1.14; 95%CI 1.01; 1.29). Longitudinal analyzes showed that age, gender, BMI, chronic diseases, physical activity, and anxiety and depression symptoms, were associated with LBP in the first pandemic months.

**Conclusion:**

Although the prevalence of LBP did not change at the first months of COVID-19 pandemic, LBP-induced impairment in daily activities and pain intensity was higher when compared to before the pandemic.

## Background

Low back pain (LBP) is one of the main musculoskeletal disorders responsible for disability [1]. The Global Burden of Disease, Injuries, and Risk Factors Study demonstrated that the prevalence of LBP increased between 1990 and 2017, especially in southern Latin America, which has the highest LBP point prevalence worldwide (13.5%) [2].

Social distancing measures were an essential strategy to avoid coronavirus disease (COVID-19) spread [3], however led to myriad changes in people’s lives. These actions, had direct and indirect effects on distinct aspects of populational health such as increasing physical inactivity and worse levels of mental health [4, 5].

Furthermore, longer time spent at home due to social distancing led to additional time spent in sedentary activities, such as sitting. The increase in those activities, as well as the decrease in active commuting due to home office, could potentially be associated with a higher burden of LBP during the pandemic [6, 7]. Also, difficulty to combine work and household activities might had a negative effect on people’s health [8].

LBP has been recognized as a health and research priority in Brazil due to its impact on productivity and days of work lost [9]. Also, the COVID-19 outbreak raised further health issues related to LBP, given an observed reduction in the number of acute LBP cases treated in clinical settings, probably as a consequence of fear to virus and disease exposure [10]. To date, no longitudinal study has investigated the effects of social distancing during the COVID-19 outbreak on LBP prevalence and related outcomes (i.e., activity limitation and pain intensity). This study aimed to evaluate how the first months of social distancing affected LBP, activity limitation, and pain intensity during the first months of pandemic.

## Materials and methods

### Study design

We analyzed data from the PAMPA cohort (Prospective Study About Mental and Physical Health), a longitudinal study designed to gather data on mental and physical health in adults living in the state of Rio Grande do Sul, southern Brazil. Data were collected on June and July of 2020, which was around three months after the first social distancing actions implemented in Brazil. By that time Brazil had an absolute and relative cases rate (per 1 M) of + 110.5 and + 109, respectively [11]. Participants answered questions related to the period before social distancing measures (retrospectively) and the current time. The study protocol was approved by the institutional research ethics board of the Faculty of Physical Education of the Federal University of Pelotas, Brazil (CAAE: 31,906,920.7.0000.5313). Details on project methods and study design can be found elsewhere [12]. The structure of the manuscript agrees with STROBE requirements.

### Sample

Sample size calculations were based on the three primary outcomes of the PAMPA Cohort (i.e., LBP, mental health, and healthcare access). The largest sample size required was 1,359 participants [13]. After accounting for a lost-to-follow-up of up to 30%, our final sample size was estimated as 1,767.

### Participant recruitment

A four-arm approach was used to achieve the target sample size [12]. Firstly, we sent information about the survey objectives and a link to access the questionnaire to researchers’ personal contacts in public and private universities within the state, asking them to spread the link. Secondly, social media campaigns (i.e., Facebook® and Instagram®) were used to deliver the questionnaire’s link to different regions of the state. Thirdly, we contacted local media (radio stations, newspapers) to inform the population about the study. Finally, each researcher involved in this survey shared the link with the questionnaire access to personal contacts across the state. The recruitment stage lasted four weeks between June and July 2020.

### Self-reported data

A self-administered online-based questionnaire was developed using the Google® Forms platform. The average time to complete the survey was approximately 10 min (range 7 to 12 min). Questions related to LBP, mental health (i.e., anxiety and depression symptoms), and physical activity were asked twice to address these outcomes at different periods (before and during social distancing).

#### LBP

We assessed LBP experience, activity limitation and pain intensity. LBP experience was assessed through an image of a person in the supine position with the low back area highlighted in a different color, followed by the question: “*Before (or During) social distancing, have you had pain in your lower back, as shown in the image, for at least one day?*”.

Pain intensity was assessed using a numeric pain rating scale where “0” indicated no pain and “10” indicated the worst pain. Activity limitation related to LBP was assessed by asking: “*Before (or During) social distancing was your low back pain severe enough to limit your daily activities for at least one day*?”.

### Exposures

Sociodemographic (i.e., gender, age, and educational level), nutritional status (i.e., Body Mass Index [BMI]), chronic diseases (e.g., Diabetes, cancer, heart disease), depressive and anxiety symptoms, physical activity, and commitment to social distancing were used as exposure variables. BMI was calculated as body weight (kg) / height (m) ^2^. Diagnostic of chronic diseases was assessed based on questions used in the Brazilian Surveillance System of Risk Factors for Chronic Diseases by Telephone Interviews (VIGITEL) [14]. Participants were also asked regarding their attitudes toward social distancing measures. For analysis purposes participants were classified based on their self-report of commitment to social distancing as follows: Low (very little and little), Medium (somewhat), and High (very much and totally isolated).

The Hospital Anxiety and Depression Scale (HDAS) was used to identify symptoms of depression and anxiety in both pre- and during social distancing [15]. This instrument includes seven items that are scored from 0 to 3, for each domain (depression and anxiety). The following criteria was used to classified the participants based on their scores: non-cases (less than 7), mild cases (between 8 and 10), moderate (between 11 and 14), and severe (between 15 and 21) [16]. As symptoms of anxiety and depression were assessed in two time points (before and during social distancing), for analyzes purposes a variable was created, regarding the change of status between timepoints, as follows: “Sustained/Better” (those who reduced or maintained their scores) or “Worse” (those who increased).

Physical activity before and during social distancing was assessed through the frequency (days per week) and time (minutes per day) participants spent practicing physical activity. A cut-off point of 150 min per week was used to classify participants as physically inactive (less than the cut-off point) or active (equal to or higher than the cut-off point), following the World Health Organization recommendation [17]. A four-category variable was created to characterize the change of physical activity status between timepoints, based on participants’ status (active or inactive) before and during social distancing, as follows: “Sustained inactive”, “Become inactive”, “Sustained active” and “Become active”.

### Data analyzes

Data were exported from Google® Sheets to Stata 15.1 (StataCorp, College Station, Texas). Due to a higher number of respondents from one mesoregion in the state (South, *N* = 1,247 [53.7%]), all analyzes were weighted for the number of respondents in each region. To verify differences between LBP and activity limitation proportions, and pain intensity levels between periods, test of difference between proportions and t-test were used, respectively. Univariate and multivariable regression analyzes were performed for LBP (Poisson Regression), activity limitation (Poisson Regression) and pain intensity (Linear Regression) to evaluate differences between time periods and their determinants. A sensitivity analysis was conducted to investigate the presence of collinearity among the anxiety, depression and physical activity variables, and four adjusted models were built. The first model was composed of sociodemographic variables (gender, age, educational level), BMI, diagnosed chronic diseases and commitment to social distancing. The second, third and fourth models were composed by the first model plus, anxiety, depression and physical activity, respectively. A *p*-value ≤ 0.20 was set to determine whether variables were kept in the model, and a p-value lower than 0.05 was adopted as the level of significance.

## Results

### Descriptive data

Participants descriptive data and LBP, before and during the first months of social distancing, are showed in Table 1. Participants with high school or lower educational levels, who were obese, reported chronic diseases, and females were more likely to experience LBP compared to their counterparts in both time periods. In addition, participants who reported worsen levels of anxiety and depression symptoms, who remained inactive before and during the pandemic, and had low adherence to social distancing measures reported higher prevalence rates of LBP in both periods analyzed.

**Table 1:** Sociodemographic, health and behavioral characteristics of participants who reported LBP. Rio Grande do Sul, Brazil, 2020 (N = 2,321)

	**Before social distancing**	**During social distancing**
Gender *(n* = *2,319)*	% (95%CI)	% (95%CI)
Male	68.1 (64.0; 71.8)	62.4 (58.1; 66.3)
Female	77.7 (75.6; 79.4)	77.2 (75.1; 79.0)
Age group *(n* = *2,300)*		
18–30	75.5 (72.9; 77.9)	73.7 (71.0; 76.1)
31–59	73.2 (70.1; 76.2)	73.3 (70.2; 76.3)
60 +	78.9 (74.3; 82.8)	73.8 (68.9; 78.1)
Educational level *(n* = *2,321)*		
High school or less	81.8 (77.4; 85.5)	82.1 (77.7; 85.7)
College degree	73.9 (71.1; 76.5)	73.3 (70.5; 75.9)
Postgraduate	74.6 (71.7; 77.2)	70.9 (68.0; 73.8)
BMI^a^*(n* = *2,315)*		
Normal	72.7 (70.0; 75.3)	70.2 (67.4; 72.9)
Overweight	77.9 (74.8; 80.6)	76.1 (73.0; 78.9)
Obese	77.6 (73.4; 81.2)	78.0 (73.9; 81.6)
Chronic disease *(n* = *2,321)*		
No	67.7 (64.8; 70.5)	69.1 (66.1; 71.7)
Yes	81.5 (79.3; 83.5)	77.5 (75.1; 79.6)
Social distancing adherence *(n* = *2,321)*		
Low	75.8 (67.5; 82.6)	81.1 (73.6; 86.9)
Middle	72.2 (66.7; 77.1)	73.4 (68.2;78.1)
High	75.2 (72.5; 77.7)	73.5 (70.7; 76.1)
Anxiety *(n* = *2,313)*		
Sustained/Better	73.8 (71.1; 76.2)	66.2 (63.4; 68.8)
Worse	77.5 (74.9; 79.7)	81.5 (79.1; 83.5)
Depression *(n* = *1,916)*		
Sustained/Better	75.4 (72.9; 77.7)	68.5 (65.7; 70.9)
Worse	76.9 (73.5; 79.9)	79.2 (75.9; 82.1)
Physical activity *(n* = *2,241)*		
Sustained inactive	78.9 (76.3; 81.3)	77.2 (74.5; 79.7)
Become inactive	72.0 (68.3; 75.4)	75.7 (72.1; 78.9)
Become active	77.9 (71.0; 83.5)	67.4 (60.0; 74.0)
Sustained active	74.0 (69.6; 77.9)	65.5 (60.9; 69.9)

^a^Body mass index

**Fig. 1:**
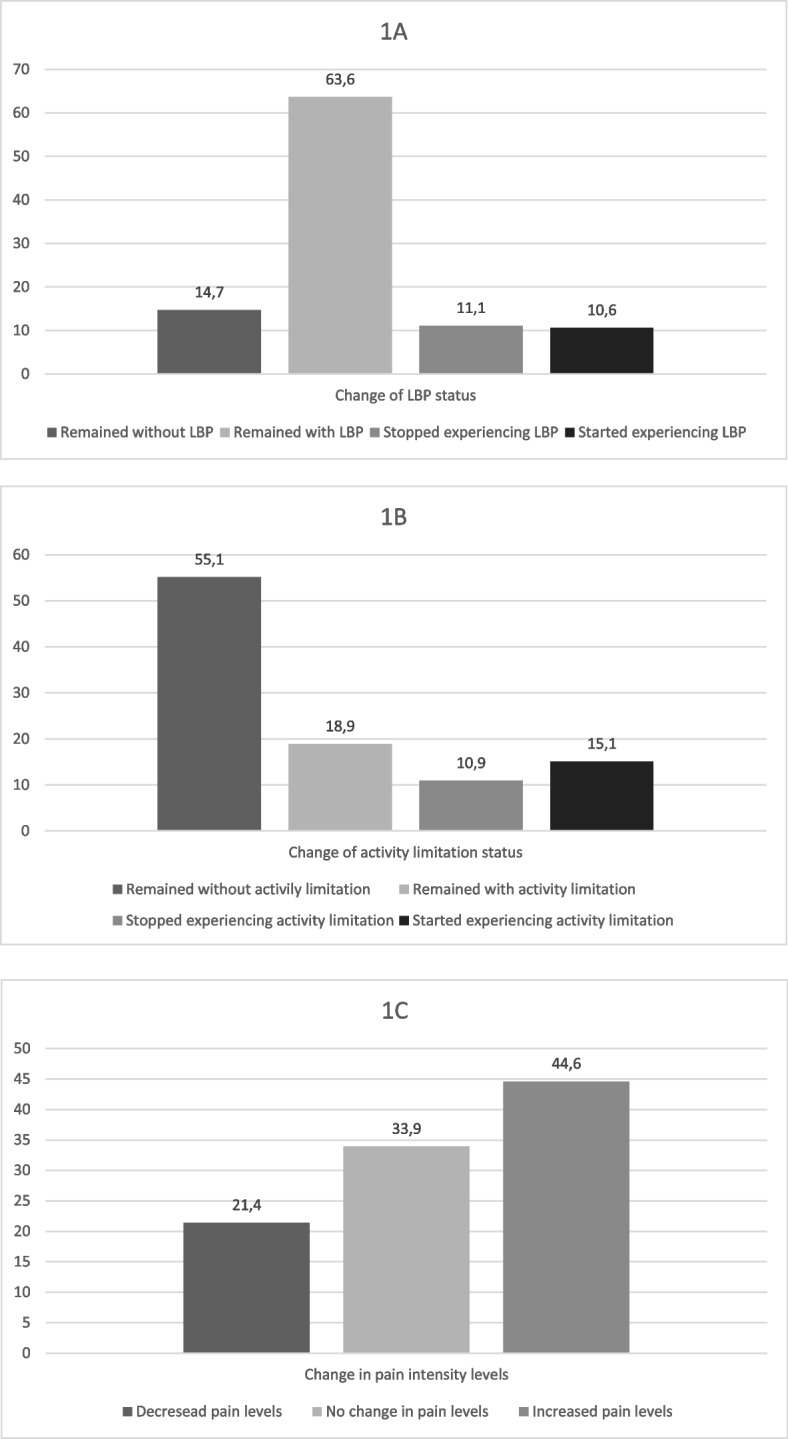
Change of LBP (1**A**), activity limitation (1**B**) and pain intensity (1**C**) status from before to during social distancing. Rio Grande do Sul, Brazil

There was no significant change of LBP prevalence from before (74.7% [95%CI 72.3; 76.9]) to during the first months of restrictions (74.2% [95%CI 71.9; 76.3]) (*p* = 0.7549). However, a significant increase in the prevalence of activity limitation was observed from 30.2% (95%CI 27.6; 32.9) to 34.1% (95%CI 31.3; 36.9) (*p *= 0.014), as well as an increased pain intensity between periods (5.3 ± 2.2 vs 5.6 ± 2.7; *p* < 0.001). Changes in LBP prevalence, activity limitation, and pain intensity status during social distancing measures are shown in Fig. 1. Most participants remained experiencing LBP during social distancing (63.6%; 95%CI 61.1; 65.9), and 10.6% (95%CI 9.1; 12.3) reported that they initiated with LBP symptoms during this period (Fig. 1A). Regarding activity limitation, 55.1% (95%CI 51.8; 58.2) of participants remained without any limitations, and 18.9% (95% CI 16.5; 21.6) reported activity limitation associated with LBP in both time periods (Fig. 1B). Also, 78.5% reported no change or an increase in pain intensity (Fig. 1C).

### Multivariable analyzes

**Fig. 2:**
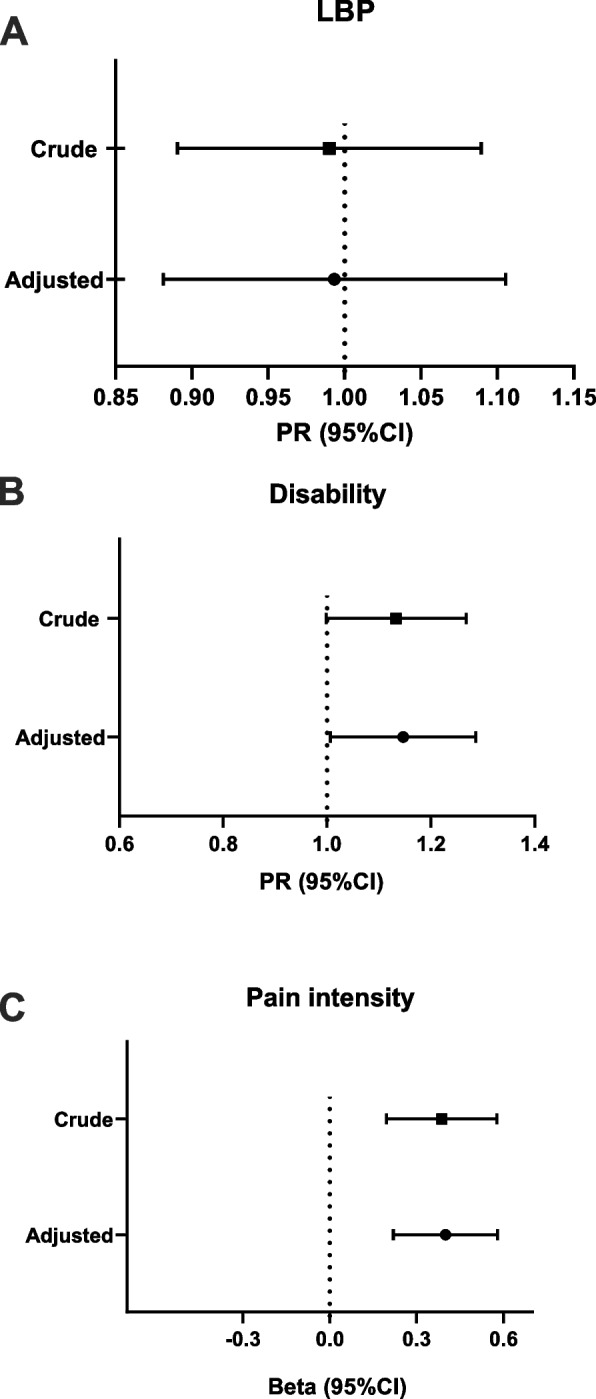
Crude and adjusted prevalence ratio (95%) for LBP (**A**) and activity limitation (**B**), and β coefficient (95%CI) for pain intensity, comparing two timepoints (before and during social distancing). Rio Grande do Sul, Brazil, 2020. LBP was adjusted for gender, educational level, BMI, chronic disease and commitment to social distancing. Activity limitation was adjusted for gender, age, chronic disease and commitment to social distancing. Pain intensity was adjusted for gender, age group, educational level, BMI and chronic disease

Regression analyzes were performed to evaluate how LBP and related outcomes changed in different groups in the two time periods. Crude and adjusted longitudinal analyzes of LBP, activity limitation and pain intensity are displayed in Fig. 2. There was no difference in the likelihood of experiencing LBP during social distancing compared to the period before (PR 0.99; 95%CI 0.95; 1.04). However, participants were 14% (95% CI 1.01; 1.29) more likely to report activity limitation during social distancing. Further, a significant increase in pain intensity was observed from before to during the pandemic (β 0.40; 95% CI 0.22; 0.58).

The factors associated with changes in LBP, activity limitation, and pain intensity between periods are shown in Tables 2, 3 and 4, respectively. Participants who were female (PR 1.16; 95%CI 1.10; 1.23), overweight (PR 1.13; 95%CI 1.08; 1.18), obese (PR 1.10 95%CI 1.04; 1.16), with diagnosed chronic diseases (PR 1.12; 95%CI 1.07; 1.17), and worsen anxiety (PR 1.06; 95%CI 1.04; 1.08) and depression (PR 1.04; 95%CI 1.02; 1.07) symptoms during pandemic were more likely to experience LBP when compared to the period before. On the other hand, participants with higher educational levels (i.e., university degree or postgraduate), were less likely to experience LBP (PR 0.91 95%CI 0.86; 0.97 and PR 0.92 95%CI 0.87; 0.97, respectively) (Table 2).

**Table 2:** Crude and adjusted longitudinal Poisson regression analyzes of LBP experience. Rio Grande do Sul, Brazil. 2020

	**Crude**		**Model 1**	
	**PR (95% CI)**	***p*-value**	**PR (95% CI)**	***p*-value**
Gender		< 0.001		< 0.001
Male	1.00		1.00	
Female	1.15 (1.09; 1.22)		1.16 (1.10; 1.23)	
Age group		0.884		0.413
18–30	1.00		1.00	
31–59	0.96 (87.4; 1.05)		1.01 (0.96; 1.06)	
60 +	0.99 (0.95; 1.03)		0.93 (0.85; 1.02)	
Educational level		0.046		0.029
High school or less	1.00		1.00	
University degree	0.90 (0.85; 0.95)		0.91 (0.86; 0.97)	
Postgraduate	0.92 (0.87; 0.97)		0.92 (0.87; 0.97)	
BMI*		< 0.001		< 0.001
Normal	1.00		1.00	
Overweight	1.12 (1.07; 1.17)		1.13 (1.08; 1.18)	
Obese	1.11 (1.05; 1.17)		1.10 (1.04; 1.16)	
Chronic disease		< 0.001		< 0.001
No	1.00		1.00	
Yes	1.15 (1.10; 1.19)		1.12 (1.07; 1.17)	
Commitment to social distancing		0.377 0.1834^a^		0.138 0.0889^a^
Low	1.00		1.00	
Medium	0.93 (0.86; 1.01)		0.92 (0.85; 1.00)	
High	0.95 (0.88; 1.01)		0.93 (0.86; 1.00)	
			**Model 2**	
Anxiety		< 0.001		< 0.001
Sustained/Better	1.00		1.00	
Worse	1.07 (1.05; 1.09)		1.06 (1.04; 1.08)	
			**Model 3**	
Depression		< 0.001		< 0.001
Sustained/Better	1.00		1.00	
Worse	1.06 (1.03;1.08)		1.04 (1.02; 1.07)	
			**Model 4**	
Physical activity		0.0313^a^		0.7289^a^
Sustained active	1.00		1.00	
Sustained inactive	1.09 (1.02; 1.16)		1.03 (0.96; 1.09)	
Become inactive	1.04 (0.97; 1.11)		1.01 (0.94; 1.09)	
Become active	1.04 (0.95; 1.14)		0.99 (0.90; 1.08)	

Model 1: adjusted for gender, educational level, BMI, chronic disease and commitment to social distancing

Model 2: Model 1 plus anxiety

Model 3: Model 1 plus depression

Model 4: Model 1 plus physical activity

^*^Body Mass Index

^a^ p for heterogeneity

**Table 3:** Crude and adjusted longitudinal Poisson regression analyzes of activity limitation. Rio Grande do Sul, Brazil. 2020

	**Crude**		**Model 1**	
	**PR (95% CI)**	***p*-value**	**PR (95% CI)**	***p*-value**
Gender		0.020		0.110
Male	1.00		1.00	
Female	1.21 (1.03; 1.42)		1.14 (0.97; 1.32)	
Age group		< 0.001		< 0.001
18–30	1.00		1.00	
31–59	1.54 (1.34; 1.79)		1.41 (1.23; 1.63)	
60 +	1.63 (1.30; 2.05)		1.30 (1.03; 1.65)	
Educational level		0.367		0.816
High school or less	1.00		1.00	
University degree	0.84 (0.71; 1.01)		0.88 (0.74; 1.05)	
Postgraduate	1.01 (0.85; 1.19)		0.94 (0.79; 1.12)	
BMI*		0.002		0.292
Normal	1.00		1.00	
Overweight	1.21 (1.04; 1.38)		1.08 (0.94; 1.25)	
Obese	1.26 (1.08; 1.48)		1.08 (0.92; 1.27)	
Chronic disease		< 0.001		< 0.001
No	1.00		1.00	
Yes	1.98 (1.71; 2.29)		1.85 (1.59; 2.15)	
Commitment to social distancing		< 0.001 0.0011^a^		0.010 0.0118^a^
Low	1.00		1.00	
Medium	1.55 (1.15; 2.08)		1.47 (1.11; 1.96)	
High	1.65 (1.26; 2.16)		1.48 (1.14; 1.93)	
			**Model 2**	
Anxiety		0.023		0.005
Sustained/Better	1.00		1.00	
Worse	1.08 (1.01; 1.15)		1.10 (1.03; 1.17)	
			**Model 3**	
Depression		< 0.001		< 0.001
Sustained/Better	1.00		1.00	
Worse	1.14 (1.07; 1.22)		1.14 (1.06; 1.22)	
			**Model 4**	
Physical activity		0.0001^a^		0.012^a^
Sustained active	1.00		1.00	
Sustained inactive	1.57 (1.29; 1.90)		1.37 (1.13; 1.65)	
Become inactive	1.37 (1.11; 1.70)		1.32 (1.07; 1.62)	
Become active	1.38 (1.03; 1.27)		1.20 (0.90; 1.29)	

Model 1: adjusted for gender, age group, chronic disease and commitment to social distancing

Model 2: Model 1 plus anxiety

Model 3: Model 1 plus depression

Model 4: Model 1 plus physical activity

^*^Body Mass Index

^a^ p for heterogeneity

**Table 4:** Crude and adjusted longitudinal Linear regression analyzes of pain intensity. Rio Grande do Sul, Brazil. 2020

	**Crude**		**Model 1**	
	***β *(95% CI)**	***p*-value**	***β *(95% CI)**	***p*-value**
Gender		< 0.001		< 0.001
Male	Ref		Ref	
Female	0.49 (0.27; 0.71)		0.50 (0.28; 0.71)	
Age group		< 0.001		0.011
18–30	Ref		Ref	
31–59	0.54 (0.35; 0.73)		0.43 (0.23; 0.64)	
60 +	0.46 (0.07; 0.84)		0.13 (-0.27; 0.53)	
Educational level		0.084		0.002
High school or less	Ref		Ref	
University degree	-0.23 (-0.48; 0.03)		-0.24 (-0.50; 0.02)	
Postgraduate	-0.27 (-0.54; -0.001)		-0.43 (-0.70; -0.16)	
BMI*		< 0.001		< 0.001
Normal	Ref		Ref	
Overweight	0.26 (0.05; 0.47)		0.21 (-0.01; 0.42)	
Obese	0.66 (0.41; 0.91)		0.48 (0.22; 0.75)	
Chronic disease		< 0.001		< 0.001
No	Ref		Ref	
Yes	0.75 (0.57; 0.94)		0.62 (0.43; 0.82)	
Commitment to social distancing		0.170 0.2161^a^		0.526 0.4578^a^
Low	Ref		Ref	
Medium	0.30 (-0.08; 0.67)		0.23 (-0.13; 0.59)	
High	0.29 (-0.04; 0.61)		0.17 (-0.15; 0.49)	
			**Model 2**	
Anxiety		< 0.001		< 0.001
Sustained/Better	Ref		Ref	
Worse	0.27 (0.18; 0.38)		0.28 (0.19; 0.38)	
			**Model 3**	
Depression		< 0.001		< 0.001
Sustained/Better	Ref		Ref	
Worse	0.23 (0.13; 0.35)		0.22 (0.11; 0.33)	
			**Model 4**	
Physical activity		< 0.001^a^		0.0002^a^
Sustained active	Ref		Ref	
Sustained inactive	0.82 (0.57; 1.07)		0.55 (0.29; 0.82)	
Become inactive	0.32 (0.04; 0.60)		0.23 (-0.05; 0.51)	
Become active	0.51 (0.11; 0.90)		0.26 (-0.15; 0.66)	

Model 1: adjusted for gender, age group, educational level, BMI and chronic disease

Model 2: Model 1 plus anxiety

Model 3: Model 1 plus depression

Model 4: Model 1 plus physical activity

^*^Body Mass Index

^a^ p for heterogeneity

Participants who were middle (PR 1.41; 95%CI 1.23; 1.63) and older-age (PR 1.30; 95%CI 1.03; 1.65), with diagnosed chronic diseases (PR 1.85 95%CI 1.59; 2.15), classified as medium/high committed with social distancing measures (PR 1.47 95%CI 1.11; 1.96, and PR 1.48; 95%CI 1.14; 1.93, respectively), and with worsened symptoms of anxiety (PR 1.10; 95%CI 1.03; 1.17) and depression (PR 1.14; 95%CI 1.06; 1.22) were more likely to report activity limitation during social distancing when compared to the pre-COVID period. Also, participants who became or sustained physically inactive during social distancing were 32% (95%CI 1.07; 1.62) and 37% (95%CI 1.13; 1.65) more likely, respectively, to have some activity limitation due to LBP (Table 3).

An increase in pain intensity was observed in participants who were female (β 0.50; 95%CI 0.28; 0.71), middle-aged (β 0.43; 95%CI 0.23; 0.64), obese (β 0.48; 95%CI 0.22; 0.75), had a chronic disease (β 0.62; 95%CI 0.43; 0.82), had worsened anxiety (β 0.28; 95%CI 0.19; 0.38) and depression (β 0.22; 95%CI 0.11; 0.33) symptoms, and sustained physically inactive (β 0.55; 95%CI 0.29; 0.82). On the other hand, a decrease in pain intensity was observed in participants with high educational level (postgraduate) (β -0.43; 95%CI -0.70; -0.16) (Table 4).

## Discussion

Our study showed similar patterns of self-reported LBP before and during the first months of pandemic in the south of Brazil. On the other hand, an increased likelihood of activity limitation and pain intensity related to LBP between periods was observed. Factors such as gender, age, educational level, BMI and diagnosed chronic diseases were related to LBP, activity limitation and higher pain intensity during the first months of social restrictions. This scenario was also related to worsen anxiety and depression symptoms, as well as to a decreased level of physical activity.

Between 2012–2016 LBP was responsible for 59 million days off work in Brazil [9]. The cost and disability from LBP were expected to increase before pandemic [1]. However, the sanitary and economic crisis installed in Brazil due to COVID-19 aggravated this scenario in short-term. Although there was a stability of self-reported LBP in the first months of social distancing compared to before, the prevalence remains high and an increase of pain intensity and activity limitation was observed.

Our study indicates that pain intensity increased less than 1 point in the first months of social restrictions, which are below the Minimal Clinically Important Difference (MCID) threshold for patients with pain [18]. However, one should note that differences in pain intensity in clinical setting, and trials, might not reflect the same impact on epidemiological studies. Also, even small increases in pain intensity might be impactful in persistent pain [19]. We revealed that two out of three participants remained with LBP in the first months of restrictions, which might be strongly affect by increased pain intensity in their daily routine.

The relationship among female gender and obesity with LBP are well stablished in literature [20, 21]. Physiological characteristics such as a decreased muscle mass may predispose women to experience LBP [22]. Also, the daily workload routine in the timpoint assessed was higher for women, as child care and household chores pile up with their paid work, which are related to this gender effect on LBP [23]. This daily overload increased during social distancing and, therefore, aggravated the burden of LBP in this population. Similarly, obesity is a known risk factor for LBP, since increasing body weight might cause an overload in the lumbar spine articular structures, which increase the risk of disk degeneration, thus reducing spinal mobility [24]. In addition, the prolonged home stay due to social distancing measures increased sedentary sitting activities, negatively affecting joint and muscles, increasing the likelihood to increase LBP.

Recent global data showed that LBP prevalence increases from 18 years onwards and peaks at the 80’s [2]. Postural problems, reduced flexibility, as well as an increased musculoskeletal degeneration are related to LBP in the aging process [25]. The decreased muscle activation during sitting position, combined with the high load on lumbar spine, can lead to LBP [26]. This is worrisome, especially during social distancing and prolonged homestay, since people spend more time sitting [27], which can consequently increase pain levels and activity limitation.

LBP is associated with several chronic diseases, such as diabetes, cancer, hypertension and pulmonary diseases [28]. Participants who reported chronic disease were more likely to experience LBP, activity limitation and high pain levels during the first months of social distancing. People with chronic diseases were instructed to stay home, and were more likely to adhere to social distancing measures, since they were in the high-risk group for COVID-19. Also, people with chronic diseases were less likely to seek in-person healthcare in the first months of pandemic [29], which can contribute to increase pain levels reported by this population.

Participants who reported worsened symptoms of depression and anxiety in the first months of social restriction were more likely to experience LBP, increased pain levels and activity limitation. We showed a sharp increase on anxiety and depression symptoms on southern Brazil population in first restriction months [30]. It has been reported that higher levels of anxiety, depression and stress are associated with physical symptoms [31]. Specifically, during COVID-19 pandemic, concerns related to lack of medical facilities or proper sanitary measures, could increase anxiety/depression and consequently increase LBP.

Studies conducted before the COVID-19 pandemic showed a protective effect of physical activity in LBP [21, 32]. However, because of social distancing and prolonged homestay, people are more likely to become or stay inactive [33–35]. Previous studies have not found an association between pain intensity and activity limitation with physical activity [36, 37]. However, it is possible that an increase in pain levels and activity limitation can be a barrier for participants to engage in physical activity, which might explain our findings.

Limitations of our study should be pointed. First, because of COVID-19 pandemic, face-to-face data collection were not allowed by ethics boards when data were collected. Thus, sampling bias cannot be ruled since internet-based surveys does not enable an equiprobable sampling since participants with low economic status are less likely to have internet access [38]. Second, the assessment of some outcomes such as disability was hampered due to online data collection. An increased time to answer the questionnaire might reduce the chance of participation, since most people uses cellphones and tablets [38]. Third, the retrospective design of our study might be subject to recall bias. However, as LBP is a remarkable event in people’s life and it has increased during COVID-19 pandemic, this bias effect is expected to be minimal. In spite of these limitations, LBP is an important health outcome and was affected by COVID-19 pandemic measures.

By the time of this paper, we have not found any large, longitudinal population-based study on the relationship between LBP and pandemic restriction measures. Also, we believe that future studies should focus on intervention strategies to reduce the LBP burden resulted from restriction measures. Strategies such as internet cognitive behavior therapy might be helpful in times of social restriction measures [39].

## Conclusion

Activity limitation and LBP intensity increased in the first months of COVID-19 pandemic in Brazil. Female sex, overweight/obese, participants who were middle-/older-age, had chronic diseases, and those who became physically inactive were more likely to experience LBP. Worsened symptoms of anxiety and depression were associated with all outcomes, which shows that mental health is highly related to LBP.

## Data Availability

The data that support the findings of this study are available from Faculty of Physical Education (Federal University of Pelotas), but restrictions apply to the availability of these data, which were used under license for the current study, and so are not publicly available. Data are however available from the authors upon reasonable request and with permission of Faculty of Physical Education (Federal University of Pelotas).

## Abbreviations

LBP: Low back pain
HDAS: Hospital Anxiety and Depression Scale
PR: Prevalence Ratio
